# New species of the genus *Ortheziola* Šulc (Hemiptera, Coccoidea, Ortheziidae)

**DOI:** 10.3897/zookeys.406.7596

**Published:** 2014-04-30

**Authors:** Mehmet Bora Kaydan, Zsuzsanna Konczné Benedicty, Éva Szita

**Affiliations:** 1 Plant Protection Institute, Centre for Agricultural Research, Hungarian Academy of Sciences, Herman Ottó u. 15 H-1022 Budapest, Hungary; 2 Çukurova Üniversity, Imamoglu Vocational School, Adana, Turkey

**Keywords:** Ensign scale, archaeococcoids, taxonomy, distribution, Palaearctic region, Oriental region

## Abstract

This paper describes three new *Ortheziola* species of the Palaearctic and Oriental regions. The specimens were extracted from forest litter using Berlese funnels, and are from the collections of Muséum d’Histoire naturelle de Genève, Switzerland. Thus the genus *Ortheziola* *sensu stricto* now includes 12 species. An identification key, distribution map and new locality records for the *Ortheziola* species currently known are provided.

## Introduction

The family Ortheziidae (Hemiptera: Coccoidea), or ensign scale insects, has been considered to be one of the oldest families in the superfamily Coccoidea (Koteja 1996, Kozár and Miller 2000, Vea and Grimaldi 2012). It is considered to be either ancestral to all scale insects, or a primitive, isolated branch of the archaeococcoid group of families. The females are distinctive, possessing well-developed legs and antennae, and having much of the body cloaked in extravagant bundles of white wax secretions, giving them a particularly flashy appearance (Vea and Grimaldi 2012). There are about 208 described species of Ortheziidae to date, in 21 genera (including four extinct genera) (Vea and Grimaldi 2012).

There are two main groups of host plant specialization in the Ortheziidae. The first group is composed of species that occur sporadically in leaf litter (presumably feeding on roots and fungal mycelia), and some are assumed to feed on mosses and lichens, habitats that are putatively the most primitive in the Coccoidea. The second species group feeds on vascular plants, including grasses, herbaceous and woody plants (Koteja 1996, Kozár 2004, Vea and Grimaldi 2012).

According to Kozár (2004), the family Ortheziidae consists of four subfamilies: Ortheziinae, Newsteadiinae, Ortheziolinae and Nipponortheziinae. The work of Vea and Grimaldi (2012) supported three of these subfamilies (Ortheziinae, Newsteadiinae and Ortheziolinae) but they found that Nipponortheziinae is not monophyletic. In species of the subfamily Ortheziolinae, the dorsum of the female is entirely covered by wax plates, and a narrow band of wax on the midline of the dorsum. This subfamily is characterized by: 3-segmented antennae (with seta size, shape, number and type very variable); eye stalk protruding, thumb-like, fused with sclerotized area at base of antenna - sometimes called the pseudobasal antennal segment; well-developed legs (trochanter and femur fused, tibia and tarsus fused; tibia with one sensory pore and often 1 or more fleshy sensory setae; tarsus without digitules; claw digitules mostly hair-like, claw without denticle); multilocular pores present (each with up to 16 outer loculi, sometimes with 1-4 central loculi, or irregularly shaped), scattered over on both dorsal and ventral surfaces; thumb-like pores forming a cluster around anal ring; abdominal spiracles ventral on anterior segments, with one present on each side of at least segments I, II, or III; if present, posterior abdominal spiracles located on dorsum near anal ring surrounded by cluster of multilocular pores; dorsum covered by wax plates.

The species of the subfamily Ortheziolinae are distributed in the Palaearctic, Oriental and Ethiopian regions, with the highest number of species in the Ethiopian region. It is divided into four tribes: Ortheziolamametini, Ortheziolini, Matileortheziolini and Ortheziolacoccini (Kozár 2004). The Ortheziolamametini occur in the Ethiopian and Oriental Regions. Members of the Ortheziolini are found in the Palaearctic Region and North-eastern part of the Oriental Region. The Ortheziolacoccini and Matileortheziolini have been found only in the Ethiopian region.

The tribe Ortheziolini is characterized by: lacking wax plates on mid-thorax; having a spine band inside the ovisac band; multilocular pores present in one row or band inside the ovisac band; and ventral setae hair-like or flagellate. This tribe contains the genus *Ortheziola* Šulc.

The most comprehensive study on the genus *Ortheziola* Šulc *sensu lato* was done by Kozár and Miller (2000); who described and assigned 16 species to the genus. Later, Kozár (2004) divided the genus and characterized *Ortheziola* *sensu stricto* by the presence of only one spine band inside the ovisac band, and by its geographical distribution. Currently, the genus *Ortheziola* *sensu stricto* contains 9 species: *Ortheziola britannica* Kozár & Miller, 2000, *Ortheziola fusiana* Shiau & Kozár, 2004, *Ortheziola marginalis* Kozár & Konczné Benedicty, 2004, *Ortheziola matskasii* Kozár & Konczné Benedicty, 2001, *Ortheziola mizushimai* Tanaka & Amano, 2007, *Ortheziola peregovitsi* Kozár & Konczné Benedicty, 2001, *Ortheziola szelenyii* Kozár & Konczné Benedicty, 1999, *Ortheziola vejdovskyi* Šulc, 1895 and *Ortheziola vietnamiensis* Kozár & Konczné Benedicty, 2001.

In this paper we describe three new *Ortheziola* species of Palaearctic and Oriental regions. All specimens were extracted from forest litter using Berlese funnels. An identification key, distribution map and new locality records for the currently known *Ortheziola* species are provided.

## Material and methods

The specimens described and recorded in this study were all collected using soil and litter sampling devices, and extracted by Berlese funnel, from samples in the Muséum d’Histoire naturelle de Genève (MHNG) collection.

Specimens were prepared for light microscopy using the slide-mounting method discussed by Kosztarab and Kozár (1988). The morphological terminology used follows Kozár (2004).

Holotypes of the new species described are deposited in the Muséum d'Histoire naturelle de Genève (MHNG). Paratypes are deposited in the MHNG and in the Plant Protection Institute, Centre for Agricultural Research, Hungarian Academy of Sciences (PPI). All measurements and counts were taken from all the material available, and the values are given as a range for each character.

## Results and discussion

### Ortheziola

Šulc, 1895

http://species-id.net/wiki/Ortheziola

#### Type species.

*Ortheziola vejdovskyi* Šulc, 1895, 1.

#### Diagnosis of genus.

Adult female in life with a series of marginal, mediolateral and medial waxy protrusions, corresponding to wax plates on slide-mounted specimens. The distribution of these protrusions (and wax plates) differs between the species (Kozár 2004).

Slide-mounted adult female with antenna 3-segmented; third antennal segment with slender apical seta, flagellate sensory seta and small subapical seta; second segment with 1 sensory pore. Eye stalk protruding, thumb-like, fused with sclerotized area at base of antenna, which is sometimes called the pseudobasal antennal segment. Legs well developed; leg setae robust, spine-like; trochanter and femur fused, tibia and tarsus fused; tibia with 1 sensory pore and at least 1 fleshy sensory seta; tarsus without digitule; claw digitules hair-like, claw without denticle. Labium 1-segmented, with many setae; with 3 long setae near apex of labium very close together, all situated in a single setal socket. Anal ring situated in a fold of derm on dorsal surface, ring bearing 6 setae. Sclerotized plate present on dorsum anterior to anal ring, wider than long. Modified pores, each with 2, 3 or 4 loculi, scattered over surface, appearing like microtubular ducts. Thumb-like pores forming cluster on each side of anal ring. Abdominal spiracles ventral on anterior segments, with at least one present on each side of segments I, II or III; when present, posterior abdominal spiracles located on dorsum near anal ring, surrounded by a cluster of multilocular pores (Kozár 2004).

#### Distribution.

The 12 species of *Ortheziola* are distributed in the Palaearctic and North East part of the Oriental Regions (Figure 4). For detailed distribution data of the nine previously known species, see ScaleNet (Ben-Dov et al. 2013). New locality records for several *Ortheziola* species were discovered during the study of the MHNG collection, which are listed below. The distribution patterns of the species may imply the existence of several other species in these regions, which would be worth further study.

#### Comments.

The genus *Ortheziola* resembles the genera *Ortheziolacoccus* and *Ortheziolamameti* in having 3-segmented antennae, and the basal part of the antenna fused to the eye. However, *Ortheziola* differs from *Ortheziolacoccus* and *Ortheziolamameti* in having only a single spine band inside the ovisac band, and by its geographic distribution. The nine previously recorded species of *Ortheziola* occur in the Palaearctic and the North-East part of the Oriental Region.

#### Key to species of *Ortheziola*, based on adult females

**Table:** 

1	Dorsal wax plates 5 and 6 present, either fused or separate	2
–	Dorsal wax plates 5 and 6 absent	10
2	Dorsal wax plate 3 present (represented by at least a small spine group)	3
–	Dorsal wax plate 3 absent	11
3	Dorsal wax plates 5 and 6 fused with marginal spine bands	*Ortheziola matskasii*
–	Dorsal wax plates 5 and 6 clearly separate from marginal spine band	4
4	Dorsal wax plate 3 reduced to a small spine group	5
–	Dorsal wax plate 3 fully developed	6
5	Ventral plate 19 present, anterior margin of ovisac band almost completely straight	*Ortheziola britannica*
–	Ventral plate 19 absent, anterior margin of ovisac band with about 8 waves	*Ortheziola marottai* sp. n.
6	Multilocular pores present around vulva	7
–	Multilocular pores absent from around vulva	8
7	Multilocular pores present both anterior and posterior to vulva; dorsal 5-locular pores present on last three abdominal segments	*Ortheziola szelenyii*
–	Multilocular pores present only anterior to vulva; dorsal 5-locular pores concentrated around anal ring	*Ortheziola vejdovskyi*
8	Ventral wax plates 11 and 19 present	*Ortheziola peregovitsi*
–	Ventral wax plates 11 and 19 absent	9
9	Ventral wax plate 12 present; marginal wax plates on abdominal segments IV–VI clearly separated from each other and from medial plates	*Ortheziola hauseri* sp. n.
–	Ventral wax plate 12 absent; marginal wax plates on abdominal segments IV–VI fused to each other and partly to medial plates	*Ortheziola mizushimai*
10	Ventral wax plates 11 and 12 present, longest seta on antenna ca. 10 µm	*Ortheziola viti* sp. n.
–	Ventral wax plates 11 and 12 absent; shortest seta on antenna ca. 19 µm long	*Ortheziola marginalis* sp. n.
11	Multilocular pores present around vulva	*Ortheziola vietnamiensis*
–	Multilocular pores absent from around vulva	*Ortheziola fusiana*

### Ortheziola hauseri

Konczné Benedicty & Kaydan sp. n.

http://zoobank.org/EDD12A42-D639-4AF3-B61F-865178DD8FC1

http://species-id.net/wiki/Ortheziola_hauseri

Fig. 1

#### Material examined.

*Holotype*. Adult female. Indonesia, North Sumatra, Prov. Mt. Sibayak, 6-7 Jul 2006, Leg. P. Schwendinger [MHNG code: Sum-6/33; PPI code: 9689].

*Paratypes*. 3 females on one slide: Indonesia, North Sumatra, Tongkoh, 1450 m a.s.l., 3 Dec 1989, Leg. Löbl, Agosti, Bruckhart [MHNG code: N Sum #29a; PPI code: 9678]; 3 females on two slides: Indonesia / North Sumatra, Brastagi, 1500 m a.s.l., 2 Dec 1989, Leg. Löbl, Agosti, Bruckhart [MHNG code: N Sum Nr. 28a; PPI code: 9679]; 1 female: Indonesia, Sumatra, Yambi, Mt. Kerinci, 1750-1850 m a.s.l., 14 Nov 1989, Leg. Löbl, Agosti, Bruckhart [MHNG code: Löbl, Agosti, Bruckhart No. 16; PPI code: 9667]; 1 female: Indonesia, Sumatra, Yambi, Tapan, 1350 m a.s.l., 9 Nov 1989, Leg. Löbl, Agosti, Bruckhart [MHNG code: Leg. Löbl, Agosti, Bruckhart No. 10; PPI code: 9670]; 2 female on one slide: Indonesia, West Sumatra, Lubuksulasih, 1100 m a.s.l., 8 Nov 1989, Leg. Löbl, Agosti, Bruckhart [MHNG: W Sum #7; PPI code: 9677]; 1 female: Indonesia, Java, Mt. Gede, 2600 m a.s.l., 5 Nov 1989, Leg. Löbl, Agosti, Bruckhart [MHNG code: W Java #5a; PPI code: 9681]; 4 females on 2 slides: Indonesia, West Java, Cibodas, 25 Nov 1987, Leg. B. Hauser [MHNG: Sar-87/14; PPI code: 9704]; 7 females on 3 slides: Malaysia / Perak, 21 Nov 1999, Leg. G. Cuccodoro, I. Löbl [MHNG: 13.MALAYSIA; PPI code: 9752].

#### Description.

*Unmounted adult female*. Not seen.

*Slide mounted adult female*. Body 1.320–1.606 mm long, 1.010–1.191 mm wide. Length of antennal segments: 1^st^ 70–102 µm; 2^nd^ 58–82 µm; 3^rd^ 250–357 µm; 3^rd^ segment parallel sided or weakly clubbed; apical seta 118–158 µm, subapical seta 36–53 µm; fleshy sensory seta near apical seta 15–22 µm; microseta present near apex of antenna; unusual hair-like seta present near subapical seta; all segments of antennae covered with moderate number of spine-like, straight, apically acute setae, longest seta 22 µm long; first antennal segment with two setae on each side of segment together with several fleshy setae.

*Venter*. Labium 120–148 µm long. Stylet loop about as long as labium. Leg segment lengths: front coxa 90–122 µm, middle 101–133 µm, hind 110–163 µm; front trochanter-femur 252–357 µm, middle 305–372 µm, hind 324–408 µm; front tibia-tarsus 316–410 µm, middle 316–439 µm, hind 382–530 µm; front claw 41–54 µm, middle 40–55 µm, hind 43–58 µm long; claw digitules spine-like, 10–22 µm long; legs with rows of robust setae; longest seta on trochanter-femur 12–17 µm; with one flagellate sensory seta on each of femur and tibia, 14–19 µm long; each trochanter with 4 sensory sensilla on each surface. Wax plates absent from marginal areas of head and thorax except for small spine cluster next to antenna (plate 12); with marginal wax band surrounding each thoracic spiracle (plates 15 and 16); without triangular-shaped wax plates in front of coxae (plates 13, 17 and 18 absent), plate 19 absent; without cluster of spines between hind legs and ovisac band; anterior edge of ovisac band with no more than 3 slight waves; with one band of spines within ovisac band. Thoracic spiracles each with scattered quadrilocular pores loosely associated with spiracle opening, each group contains 32–36 pores, each pore 4 µm in diameter (several of these pores present on dorsum); diameter of opening of anterior thoracic spiracle 20–24 µm. Setae few, scattered in medial areas of thorax, with several setae present near anterior edge of ovisac band (some capitate), several associated with anterior and posterior multilocular pore rows, several more associated with posterior multilocular pores surrounding vulva. Multilocular pores each 7 µm in diameter, with 8–12 loculi around perimeter and one loculus in central hub; with quadrilocular pores predominant near anterior edge of spine band, partial row of multilocular pores near anterolateral edge of spine band, also scattered around vulva and near ovisac band, almost forming a row on the apical abdominal segment. Abdominal spiracles present, 2 pairs on each side of body anterior to ovisac band and one pair situated inside ovisac band, near anterolateral angle; each abdominal spiracle with sclerotized vestibule.

*Dorsum*. Wax plates covering two-thirds of marginal area; mediolateral thoracic plates (3, 5 and 6) absent; medial area of thorax and abdomen without spines or pores. Spines at margin of wax plate 4 each 15 µm long, in middle of wax plate each 15–18 µm long; spines truncate and expanded at apex. Flagellate setae present in marginal clusters near posterior edges of marginal wax plates (2 and 4), with 2–4 setae lateral to each thoracic spiracle, each seta 20 µm long; also present in very small numbers on other wax plates and in medial bare area; blunted setae present in the middle area of the body, each 8 µm long. Quadrilocular pores, each 4 µm in diameter, with 4 loculi, present in marginal areas of abdomen; also present in cluster near anal ring, the pores in this cluster sometimes each with 5 loculi. Sclerotized plate on abdomen 70–80 µm long, 200–260 µm wide; several setae situated at posterior edge of plate, many with capitate apices. Anal ring with incomplete triple row of circular pores, each pore 2–3 µm in diameter; longest anal ring seta 40–60 µm long (about same length as anal ring); anal ring 40–51 µm wide. Thumb-like pores each 5–6 µm long. Modified pores each 5–7 µm long. Abdominal spiracle present in centre of multilocular pore cluster situated laterad of anal ring.

**Figure 1.**
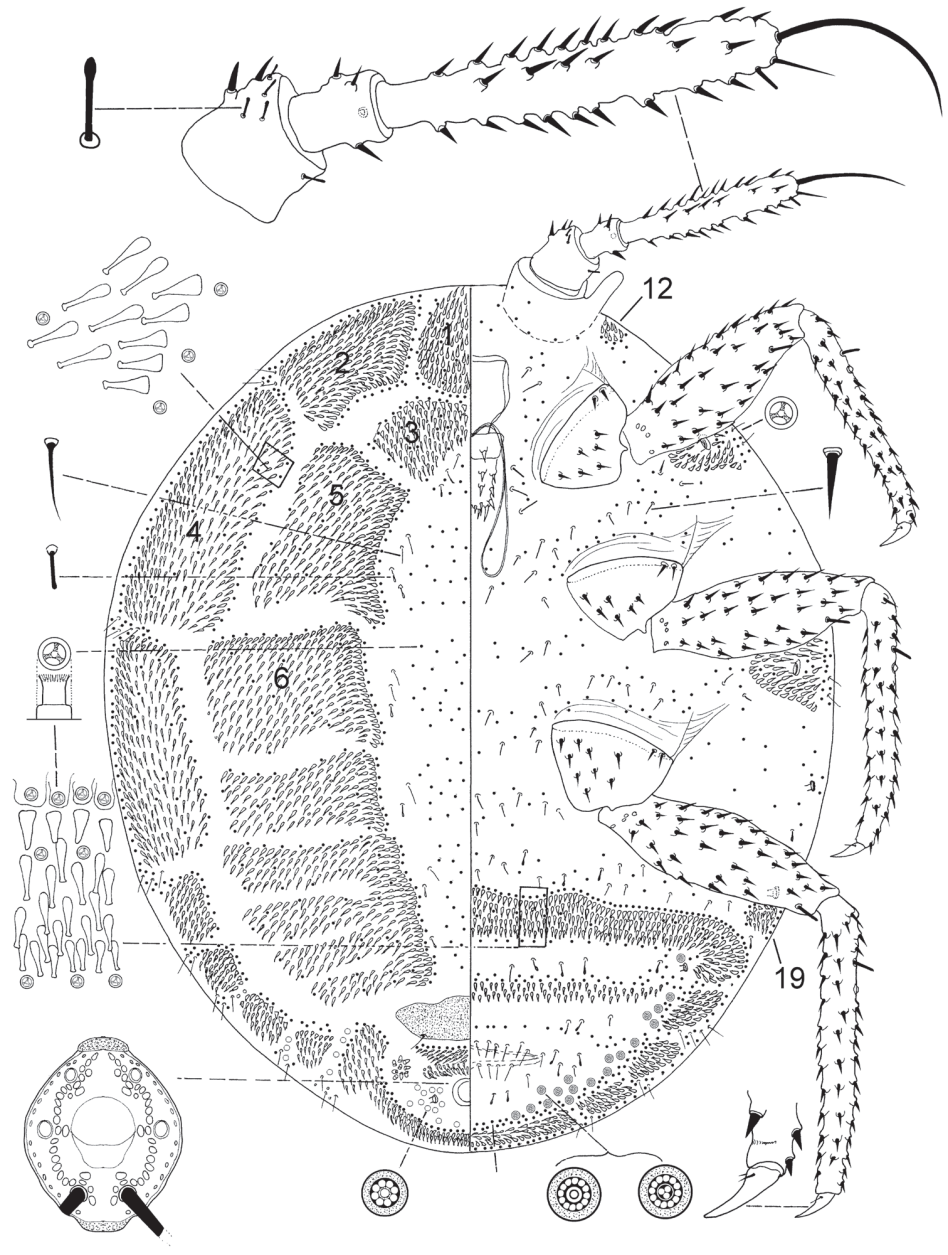
*Ortheziola hauseri* Konczné Benedicty & Kaydan sp. n., holotype, adult female.

#### Host plant.

Unknown.

#### Distribution.

Indonesia, Malaysia (Fig. 4).

#### Etymology.

The new species is named after B. Hauser, as his record from 1987 was the oldest in the type series.

#### Comments.

*Ortheziola hauseri* is characterized by having dorsal wax plate 3 fully developed, and ventral plates 11 and 19 absent from near the body margin. This species is very close to *Ortheziola vejdovskyi* but differs by having (*Ortheziola vejdovskyi* values in brackets): i.) long spines on antenna, the longest 22 µm (12–16 µm); ii.) absence of ventral plate 11 (present) and iii.) multilocular pores absent from around vulva (present).

### Ortheziola marottai

Kaydan & Szita sp. n.

http://zoobank.org/94ADD9F9-D78B-4513-944E-A264D97F8944

http://species-id.net/wiki/Ortheziola_marottai

Fig. 2

#### Material examined.

*Holotype*. Adult female. Greece / Thessaly, 08 Apr 2004, Leg. S. Vit [MHNG code: GR-2004 No.1; PPI code: 8892-signed red].

*Paratypes*. 1 female on same slide as holotype; 3 females on two other slides: Greece, Thessaly, Peloponessos, Gorge de Kalvrita, 3 Apr 1971, Leg. I. Löbl [MHNG code: GR-71/75; PPI code: 8891]; 1 female: Greece, Lania / 11 Jun 1980, Leg. J.T. Huber [MHNG code: -; PPI code: 8889]; 2 females: Greece, Peloponessos, 1975, Leg. B. Hauser [MHNG code: Hel-75/1, PPI code: 8917].

*Other material examined*. 1 female: Croatia (Former Yugoslavia), Dalmatia, 1 Aug 1976 Leg. P. Strinati [MHNG code: Ju-76/2; PPI code: 8929]; 1 female: Turkey / İzmir, Çeşme, 17 Sep 1988, Leg. T. Jaccoud et al. [MHNG code: TR 234; PPI code: 8956); 1 female, Cyprus, Leg. S. Vit, 13.iv.1998 [MHNG code: 98/19B; PPI code: 8959]; 2 females on one slide: Iran, Gilān, Pareh-Sar, leaf litter at hollow tree / 2 Jul 1973, Leg. A. Senglet [MHNG code: 7310 Iran; PPI code: 8932].

#### Description.

*Unmounted adult female*. Not seen.

*Slide mounted adult female*. Body 1.631–1.917 mm long, 1.1921–1.554 mm wide. Length of antennal segments: 1^st^ 79–89 µm; 2^nd^ 60–68 µm; 3^rd^ 276–326 µm; 3^rd^ segment parallel sided or weakly clubbed; apical seta 115–148 µm long, subapical seta 40–48 µm long; fleshy sensory seta near apical seta 15–20 µm long; microseta present near apex of antenna; unusual hair-like seta present near subapical seta; all segments of antennae covered with moderate number of spine-like, straight, apically acute setae, longest seta 10 µm long; first antennal segment with one seta on each side of segment.

*Venter*. Labium 173 µm long. Stylet loop about as long as labium. Leg segment lengths: front coxa 98–128 µm, middle 101–133 µm, hind 120–149 µm; front trochanter-femur 360–408 µm, middle 380–421 µm, hind 384–449 µm; front tibia-tarsus 383–408 µm, middle 391–440 µm, hind 384–534 µm; front claw 52–60 µm, middle 52–55 µm, hind 56–60 µm long; front claw digitules 5 µm long, middle 5 µm long, hind 5–14 µm long; legs with rows of robust setae; longest on trochanter-femur 10 µm; with one flagellate sensory seta on tibia, 15–25 µm long; each trochanter with 4 sensory sensilla on each surface. Wax plates absent from marginal areas of head and thorax except for small spine cluster next to antenna (plate 12) and normal plate between antennae (plate 11), with marginal wax band surrounding each thoracic spiracle (plates 15 and 16); without triangular-shaped wax plates in front of coxae (plates 13, 17, 18 and 19); without cluster of spines between hind legs and ovisac band; anterior edge of ovisac band with about 8 waves; with one band of spines within ovisac band. Thoracic spiracles each with scattered quadrilocular pores loosely associated with spiracular opening, each group containing 28–42 pores, each pore 7 µm in diameter (several of these pores on dorsum); diameter of opening of anterior thoracic spiracle 22 µm. Setae few, scattered in medial areas of thorax, with several setae near anterior edge of ovisac band (some capitate), several associated with anterior and posterior multilocular pore rows, several more associated with posterior multilocular pores surrounding vulva. Multilocular pores each 6–10 µm in diameter with 4–11 loculi around perimeter, and one loculus in central hub;with quadrilocular pores predominant near anterior edge of spine band, partial row of multilocular pores near anterolateral edge of spine band, also scattered around vulva and near ovisac band, almost forming a row on the apical abdominal segment. Abdominal spiracles present with 3 pairs on each side of body anterior of ovisac band and one pair situated inside ovisac band, near anterolateral angle; each abdominal spiracle with sclerotized vestibule.

*Dorsum*. Wax plates covering two-thirds of marginal area; mediolateral thoracic plates small (plates 3, 5 and 6), covering most of mediolateral thoracic areas; plate 3 divided medially; medial area of thorax and abdomen without spines or pores. Spines at margin of wax plate 4 each 13–15 µm long, in middle of wax plate each 15–18 µm long; spines truncate and expanded at apex. Flagellate setae present in marginal clusters near posterior edges of marginal wax plates (plates 2 and 4), with 2–4 setae lateral of each thoracic spiracle, each 22 µm long; also present in very small numbers on other wax plates and in medial bare area. Multilocular pores each 6–7 µm in diameter, with 4 loculi, present in marginal areas of abdomen; also present in cluster near anal ring, the pores in this cluster sometimes each with 5 loculi. Sclerotized plate on abdomen 55–70 µm long, 242–290 µm wide; several setae situated at posterior edge of plate, many with capitate apices. Anal ring with incomplete triple row of circular pores, each pore 2–3 µm in diameter; longest anal ring seta 45–60 µm long, about equal to length of anal ring, which is 48–58 µm wide. Thumb-like pores each 5–6 µm long. Modified pores each 5–7 µm long. Abdominal spiracle present in centre of multilocular pore cluster situated laterad of anal ring.

**Figure 2.**
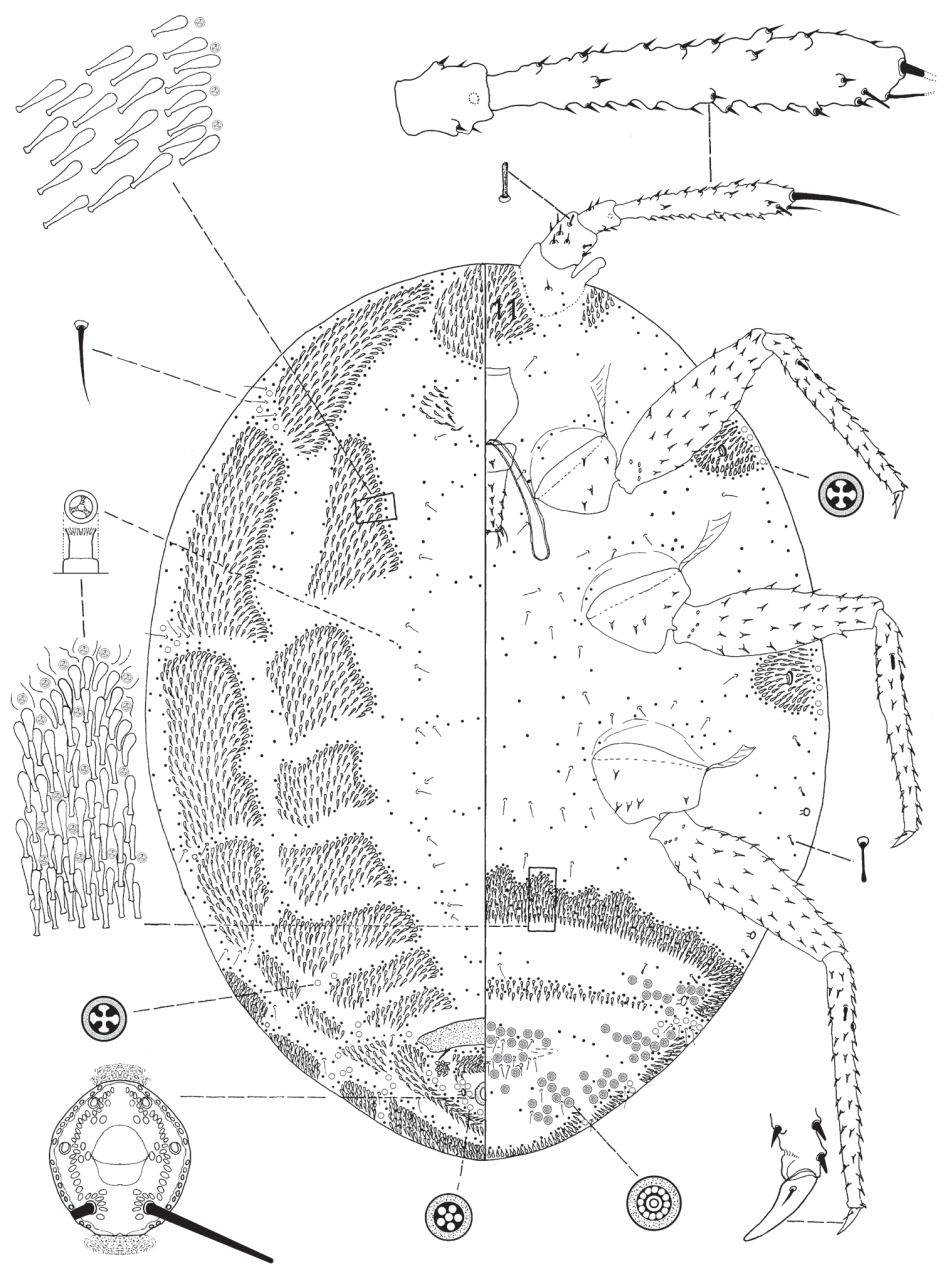
*Ortheziola marottai* Kaydan & Szita sp. n., holotype, adult female.

#### Host plant.

Unknown.

#### Distribution.

Croatia (former Yugoslavia), Cyprus, Greece, Iran, Turkey (Fig. 4).

#### Etymology.

The new species is named after the Italian coccidologist, Salvatore Marotta (Catania University, Italy).

#### Comments.

*Ortheziola marottai* is characterized by having dorsal wax plate 3 divided medially, and lacking ventral plate 19 near the body margin. This species very close to *Ortheziola britannica* but differs by having (*Ortheziola britannica* values in brackets): i.) anterior margin of ovisac band wavy (anterior margin of ovisac band straight); ii.) large numbers of multilocular pores around vulva and on abdominal segments (multilocular pores only present in small numbers) and iii.) total absence of ventral plate 19 (plate 19 present).

### Ortheziola viti

Szita & Konczné Benedicty sp. n.

http://zoobank.org/BA49391E-64D9-47E7-8A3C-9534305BB902

http://species-id.net/wiki/Ortheziola_viti

Fig. 3

#### Material examined.

*Holotype*. Adult female. Greece, Thessaly, North-Tsakgarada, 550 m a.s.l., in hollow base of *Platanus* sp., 09 Apr 2004, Leg. S. Vit [MHNG: GR-2004 No.3; PPI code: 8915].

*Paratypes*. 2 adult females, 1 specimen on same slide as holotype, 1 specimen on separate slide, same data as holotype [MHNG code: GR-2004 No.3; PPI code: 8915]; 4 females on 3 slides: Turkey, North Elma-Dagi, Ankara-slopes, 1200 m a.s.l., *Crataegus* sp. litter, 31 Oct 1995 Leg. S. Vit [MHNG code: ANK. No.3; PPI code: 9463].

#### Description.

*Unmounted adult female*. Not seen.

*Slide mounted adult female*. Body 1.320–1.476 mm; 1.030–1.192 mm wide. Length of antennal segments: 1^st^ 60–75 µm; 2^nd^ 36–51 µm; 3^rd^ 208–228 µm; 3^rd^ segment parallel sided or weakly clubbed; apical seta 91–120 µm long, subapical seta 32–40 µm long; fleshy sensory seta near apical seta 11–14 µm long; microseta present near apex of antenna; unusual hair-like seta present near subapical seta; all segments of antennae covered with moderate number of spine-like, straight, apically acute setae, longest seta 10 µm long; first antennal segment with one seta on each side of segment.

*Venter*. Labium 117–136 µm long. Stylet loop about as long as labium. Leg segment lengths: front coxa 90–102 µm, middle 97–107 µm, hind 120–140 µm; front trochanter-femur 235–244 µm, middle 240–276 µm, hind 281–302 µm; front tibia-tarsus 250–269 µm, middle 269–282 µm, hind 326–355 µm; front claw 40–47 µm, middle 40–44 µm, hind 44–48 µm long; claw digitules spine-like, 5–6.5 µm long; legs with rows of robust setae; longest seta on trochanter-femur 10–12 µm; with one flagellate sensory seta on tibia, 21 µm long; each trochanter with 4 sensory sensilla on each surface. Wax plates absent from marginal areas of head and thorax except for small spine cluster next to antenna (plate 12) and normal plate between antennae (plate 11), and with marginal wax band surrounding each thoracic spiracle (plates 15 and 16); without triangular-shaped wax plates in front of coxae (plates 13, 17 and 18); plate 19 absent; without cluster of spines between hind legs and ovisac band; anterior edge of ovisac band almost completely straight; with one band of spines within ovisac band. Thoracic spiracles each with scattered quadrilocular pores loosely associated with spiracle opening, each group containing 20–28 pores, each pore 4 µm in diameter (several of these pores present on dorsum); diameter of opening of anterior thoracic spiracle 18–22 µm. Setae few, scattered in medial areas of thorax, with several setae near anterior edge of ovisac band (some of them capitate), several associated with anterior and posterior multilocular pore rows, several more associated with posterior multilocular pores surrounding vulva. Multilocular pores each 7 µm in diameter, with 8–12 loculi around perimeter and one loculus in central hub; with quadrilocular pores predominant near anterior edge of spine band, partial row of multilocular pores near anterolateral edge of spine band, also scattered around vulva and near ovisac band, almost forming a row on the apical abdominal segment. Abdominal spiracles present with 2 pairs on each side of body anterior of ovisac band and one pair inside ovisac band, near anterolateral angle; each abdominal spiracle with sclerotized vestibule.

*Dorsum*. Wax plates covering two-thirds of marginal area; mediolateral thoracic plates absent (plates 3, 5 and 6); medial area of thorax and abdomen without spines and pores. Spines at margin of wax plate 4 each 17–18 µm long, in middle of wax plate each 15–18 µm long; spines truncate and expanded at apex. Flagellate setae present in marginal clusters near posterior edges of marginal wax plates (plates 2 and 4), with 2–4 setae lateral of each thoracic spiracle, each 18–20 µm long; also present in very small numbers on other wax plates and in medial bare area. Multilocular pores, each 4 µm in diameter, with 4 loculi, present in marginal areas of abdomen; also present in cluster near anal ring, the pores in this cluster sometimes each with 5 loculi. Sclerotized plate on abdomen 45–60 µm long, 190–240 µm wide; several setae situated at posterior edge of plate, many with capitate apices. Anal ring with incomplete triple row of circular pores, each pore 2–3 µm in diameter; longest anal ring seta 43–49 µm long (about equal to length of anal ring); anal ring 47–54 µm wide. Thumb-like pores each 5–6 µm long. Modified pores each 5–7 µm long. Abdominal spiracle in centre of multilocular pore cluster situated laterad of anal ring.

**Figure 3.**
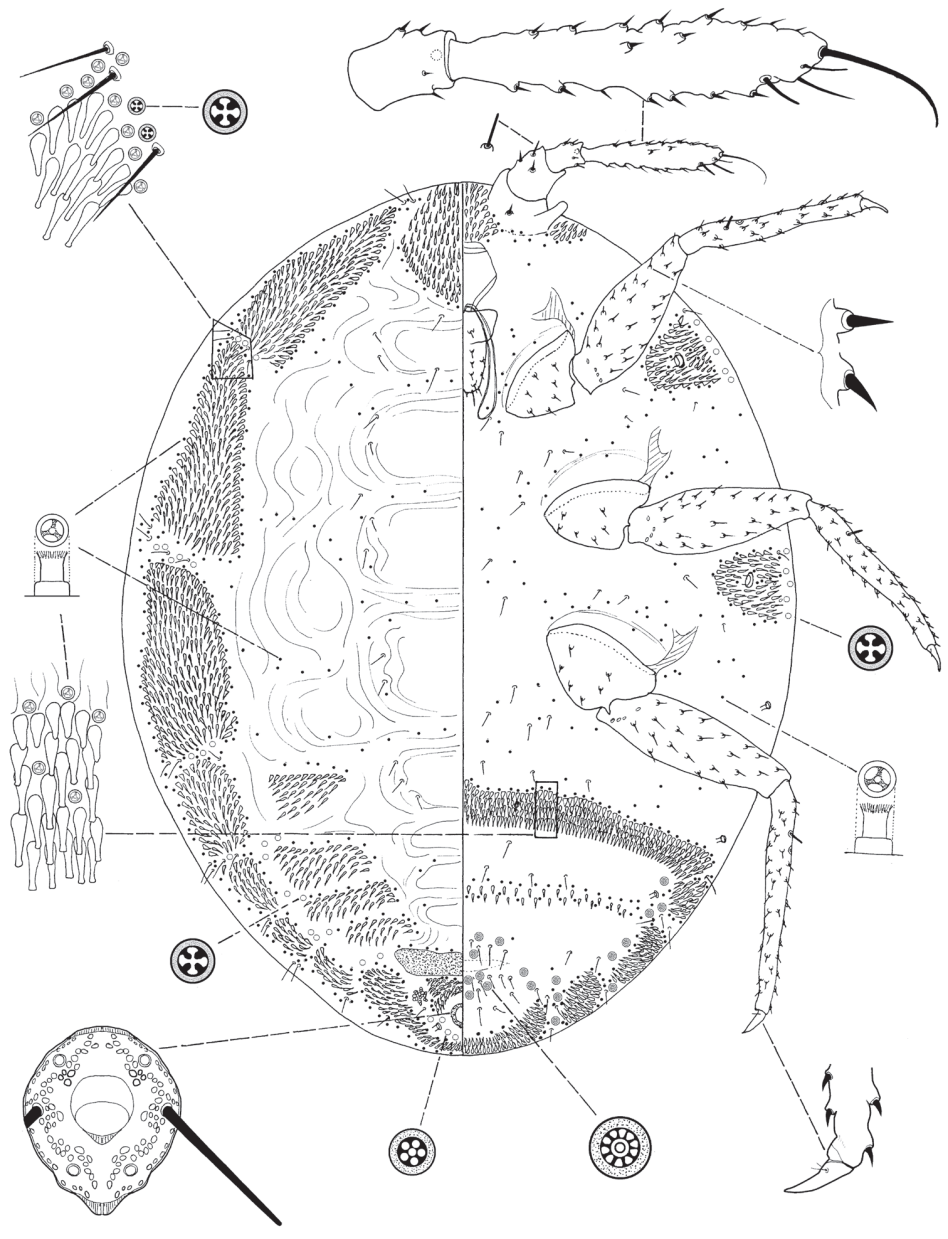
*Ortheziola viti* Szita & Konczné Benedicty sp. n., holotype, adult female.

#### Host plant.

Unknown.

#### Distribution.

**Figure 4.**
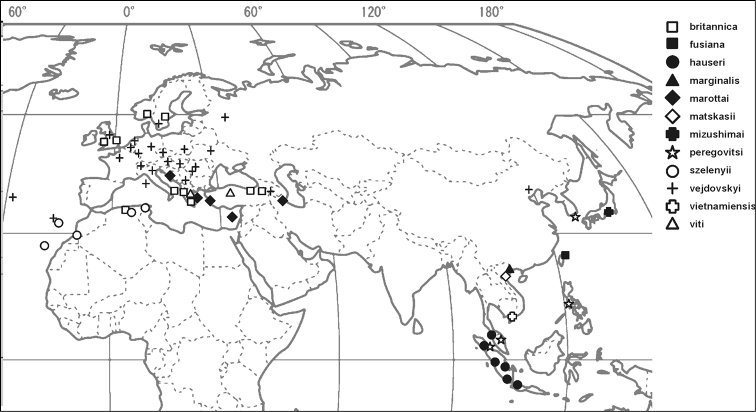
Distribution map of *Ortheziola* species.

Greece, Turkey (Fig. 4).

#### Etymology.

The new species is named after S. Vit, the collector of the type series.

#### Comments.

*Ortheziola viti* is characterized by having dorsal wax plate 3 divided medially; ventral plate 19 absent from near the body margin, and dorsal plates 3, 5 and 6 absent. This species is very close to *Ortheziola marginalis* but differs by having (character in brackets belongs to *Ortheziola marginalis*): i.) short spines on antenna, the longest 10 µm (shortest 19 µm); ii.) plates on abdominal segments III-VII all divided (plates on abdominal segments III-VII not divided); and iii.) ventral plates 11 and 12 present (absent).

### Distribution of *Ortheziola* species in the world

***Ortheziola britannica* Kozár & Miller, 2000**

**Distribution.** Currently recorded from Great Britain, Italy and Sweden (Ben-Dov et al. 2013). New records: Algeria, Blida, Mts. Atlas, 1400 m a.s.l., 3 May 1988, Leg. Besuchet, Löbl & Burckhardt [MHNG code: 1d; PPI code: 8960]; Greece, Epirus, Perdika, 27.iii.1978, Leg. S. Vit [MNHG code: 17Grece; PPI code: 8918]; Greece, Epirus, Asproblision?, 5 May 1973, Leg. I. Löbl [MNHG code: EP-73/94; PPI code: 8919]; Greece, Peloponessos, 113 m. a.s.l., 20.iv.1975, Leg. B. Hauser [MNHG code: Hel-75/7; PPI code: 8921]; Norway, Bamble, N Langøya hovedgard, Telemark, UTM 32vnl4315341168, 24 Jun 2009, Leg. Olsen [MNHG code: EIS11; PPI code: 9260]; Turkey, Kars, 16 km South-East of Göle, 1600 m a.s.l., 16 Jun 1986, Leg. Besuchet, Löbl & Burckhardt [MNHG code: 24d; PPI code: 8880]; Turkey, Gümüshane, 30 km of Erzincan, 2100 m a.s.l., 4 Jun 1986, Leg. Löbl, Besuchet & Burckhardt [MNHG: 1a; PPI code: 8883].

***Ortheziola fusiana* Shiau & Kozár, 2004**

**Distribution.** Taiwan (Ben-Dov et al. 2013).

***Ortheziola marginalis*** Kozár & Konczné Benedicty, 2004

**Distribution.** Vietnam (Ben-Dov et al. 2013).

***Ortheziola matskasii*** Kozár & Konczné Benedicty, 2001

**Distribution.** Vietnam (Ben-Dov et al. 2013).

***Ortheziola mizushimai*** Tanaka & Amano, 2007

**Distribution.** Japan (Ben-Dov et al. 2013).

***Ortheziola peregovitsi* Kozár & Konczné Benedicty, 2001**

**Distribution.** Currently recorded from South Korea (Ben-Dov et al. 2013). New records: Indonesia, Sumatra, Utara, Deli Serdang, 19.11.1985, Leg. B. Hauser [MNHG code: Sum-85/47; PPI code: 9826]; Malaysia, Pahang, Beringin Beach Resort, 21 Nov 2001, Leg. I. Löbl [MNHG: Malaisie (Pahang) and i96; PPI code: 9823, 9824]; Philippines, Mindoro, 27-29.12.1979, Leg. Deharveng & Crousset [MNHG code: Malaisie (Pahang); PPI code: 9828].

***Ortheziola szelenyii* Kozár & Konczné Benedicty, 1999**

**Distribution.** Currently recorded from Tunisia (Ben-Dov et al. 2013). New records: Algeria, 4 Apr 1971, Leg. J. Steffen [MNHG code: 4/201; PPI code: 8951]; Morocco, Imouzzèr, Cascades d’Imouzzèr, 11 May 1974, Leg. C. Besuchet [MNHG code: Mar-74/9; PPI code: 8952]; Portugal, Madeira, Rosario, south of Sao Vincente, 1000 m a.s.l., 22.05.1984, Leg. P. Hozman [MNHG: -; PPI code: 8887]; Spain, Canary Islands, Hierro, El Golfo, sous le Mirador de Jinama, 8.03.1983 /Leg. C. Besuchet [MNHG: -; PPI code: 8931]; Spain, Canary Islands, Tenerife, Mt. De las Mercedes, 1000 m a.s.l., 23.04.1976, Leg. S. Vit [MNHG: -; PPI code: 8934].

***Ortheziola vejdovskyi* Šulc, 1895**

**Distribution.** Armenia, Austria, Belgium, Bulgaria, China, Czech Republic, France, Germany, Greece, Great Britain, Hungary, Italy, Luxembourg, The Netherlands, Poland, Portugal, Romania, Sweden, Switzerland, Ukraine, USSR (former), Yugoslavia (former) (Ben-Dov et al. 2013).

***Ortheziola vietnamiensis* Kozár & Konczné Benedicty, 2001**

**Distribution.** Vietnam (Ben-Dov et al. 2013).

## Supplementary Material

XML Treatment for Ortheziola

XML Treatment for Ortheziola hauseri

XML Treatment for Ortheziola marottai

XML Treatment for Ortheziola viti
